# Effects of Green Color Exposure on Stress, Anxiety, and Pain during Peripheral Intravenous Cannulation in Dental Patients Requiring Sedation

**DOI:** 10.3390/ijerph18115939

**Published:** 2021-06-01

**Authors:** Yukihiko Takemura, Kanta Kido, Hiromasa Kawana, Tatsuo Yamamoto, Takuro Sanuki, Yoshiharu Mukai

**Affiliations:** 1 Department of Restorative Dentistry, Kanagawa Dental University, Yokosuka 2388580, Kanagawa, Japan; takemura@kdu.ac.jp (Y.T.); mukai@kdu.ac.jp (Y.M.); 2 Department of Dental Anesthesiology, Kanagawa Dental University, Yokosuka 2388580, Kanagawa, Japan; sanuki@kdu.ac.jp; 3 Department of Oral and Maxillofacial Implantology, Kanagawa Dental University, Yokosuka 2388580, Kanagawa, Japan; kawana@kdu.ac.jp; 4 Department of Dental Sociology, Kanagawa Dental University, Yokosuka 2388580, Kanagawa, Japan; yamamoto.tatsuo@kdu.ac.jp

**Keywords:** anxiety, pain, salivary alpha-amylase, peripheral intravenous cannulation, green color exposure, visual analog scale

## Abstract

Intravenous cannulation is an invasive procedure that causes stress, anxiety, and pain for many patients. A recent animal study found that exposure to green light induced antinociceptive and anxiolytic effects. This study examined whether green color exposure reduced stress, anxiety, and pain during peripheral intravenous cannulation (PIC) for sedation in dental patients. In this controlled clinical trial, 24 patients (mean age 40.9 years) were randomized to wear clear glasses or green-colored glasses for 15 min before PIC on two separate days in a cross-over manner. The primary outcome measures were salivary alpha-amylase (sAA) activity and stress-related hemodynamic changes, and the secondary outcome measures were the visual analog scale anxiety (VAS-A) and pain (VAS-P) scores during PIC. The sAA level in the clear group significantly increased during PIC compared with baseline, but did not increase in the green group. Median VAS-P scores during PIC were lower in the green group than in the clear group (VAS-P, 17.0 vs. 50.0). Green color exposure with glasses significantly reduced stress and pain during PIC without any adverse effects. This simple, safe, and effective method may be useful during painful medical procedures.

## 1. Introduction

Intravenous (IV) cannulation is an invasive procedure that provokes stress, pain, anxiety, and fear in patients [1,2,3,4]. These unpleasant feelings can have adverse implications for future treatment and care of patients, so appropriate pain and anxiety management is of paramount importance [5,6]. Pharmacological and nonpharmacological interventions to reduce the pain and anxiety associated with peripheral IV cannulation (PIC) have been described [1,7,8]. Although local anesthesia injection, local anesthetic cream, and vapocoolant spray are widely reported to reduce pain during PIC [9,10,11], these strategies target pain but do not address anxiety and fear. Furthermore, they are not often used because they are expensive, time-consuming, and prone to causing allergic reactions or vasoconstriction and may be hard to use [5,12,13]. Therefore, simple, safe, easy-to-use, non-invasive, cost-effective, and reusable methods have been recommended [14].

Ibrahim et al. recently reported that exposure to green light resulted in antinociception and anti-hyperalgesia in rats and depended on engagement of the visual system [15]. In that study, green light exposure also induced an anxiolytic effect. Moreover, the same research group showed that green light improved pain and quality of life in patients with fibromyalgia and migraine [16,17]. Therefore, green color exposure before a medical procedure might reduce pain and anxiety during PIC.

IV sedation allows patients with phobia or a gag reflex to receive necessary dental treatment. However, in one study, 42.9% of such patients were anxious about a painful procedure and 46.0% had a fear of needles [18]. In other words, these patients may be more sensitive to fear and pain during PIC, so that they could need more effective pain management.

Therefore, this study examined whether green color exposure using glasses before PIC can reduce stress, anxiety, and pain during PIC in dental patients requiring IV sedation. We evaluated salivary alpha-amylase (sAA) activity and hemodynamic changes as objective measures and the visual analog scale score for anxiety (VAS-A) and visual analog scale score for pain (VAS-P) as subjective measures.

## 2. Materials and Methods

### 2.1. Study Design and Setting

**Figure 1:**
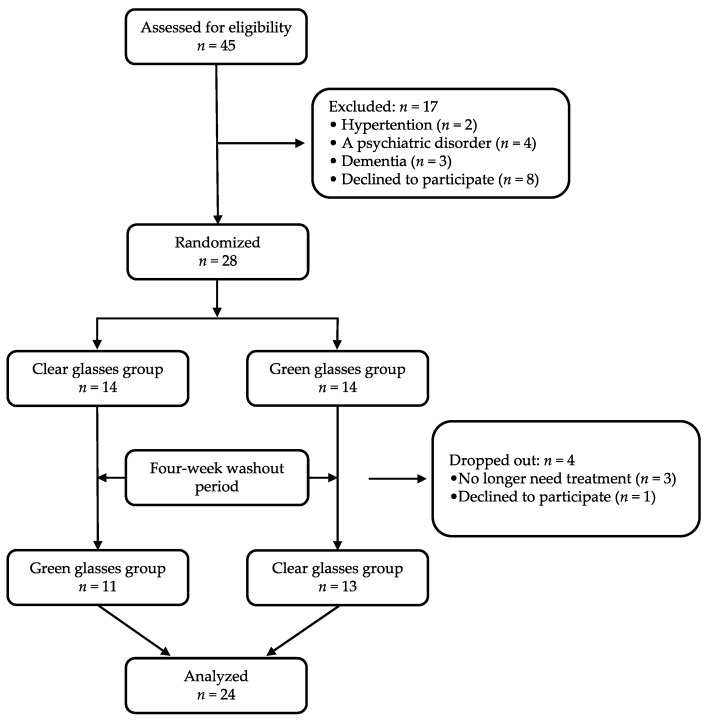
Study flow chart. Recruitment and allocation to study groups.

This randomized, controlled, single-center, cross-over clinical trial was conducted between April 2019 and March 2020 at the Kanagawa Dental University Hospital, Yokosuka, Japan (Figure 1).

### 2.2. Study Population

Adult patients (aged older than 20 years) undergoing IV sedation for dental treatment were included. The exclusion criteria were chronic use of analgesic or psychiatric medication, history of color blindness or uncorrected cataracts, severe cardiovascular or respiratory disease, an inflammatory disorder, and communication difficulties. Patients in whom the first PIC attempt was unsuccessful were also excluded.

### 2.3. Procedure

All study participants received an explanation of the purpose of the study during the preoperative consultation. The patients underwent two color exposures with glasses (Color Therapy Glasses, GloFX, FL, USA) on two different days: wear of green-colored glasses or clear-color glasses (as control). Using a computer-generated randomization method, the participants were randomly allocated to PIC with green color exposure (green group) or clear color exposure (clear group) first, and then crossed over to the other type of glasses after a 4-week washout period. It was not possible to blind the participants to group allocation. Therefore, to minimize bias and expectations, the patients were not told which color affected stress, anxiety, and pain during PIC.

**Figure 2:**
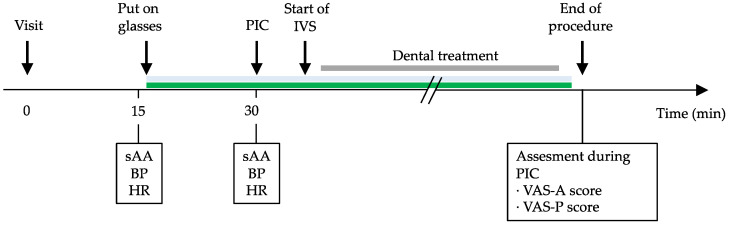
Study protocol. BP, blood pressure; HR, heart rate; PIC, peripheral intravenous cannulation; sAA, salivary alpha-amylase; VAS-A, visual analog scale for anxiety; VAS-P, visual analog scale for pain.

The patients were not allowed to drink for 3 h or eat for 6 h before treatment to avoid vomiting during IV sedation. After 15 min of rest in a waiting room, baseline values for salivary amylase activity (sAA) and vital signs, including systolic blood pressure (SBP), diastolic blood pressure (DBP), mean blood pressure (MBP), and heart rate (HR), were measured. The patients then put on their trial glasses and stayed in the waiting room for 15 min, after which they entered the treatment room and were seated in the dental chair. The patients in both groups were treated by a clinician with at least 5 years of experience performing PIC. While the patient’s blood pressure (BP) and HR were monitored, IV cannulation was performed with a 22- or 24-gauge catheter via a vein on the dorsum of the hand. The sAA level and vital signs were then measured and recorded during PIC. Dental treatment was started when sedation was confirmed. After the procedure, when the patients had recovered from sedation, they rated their feelings during PIC using the VAS-A and VAS-P (Figure 2).

### 2.4. Outcome

#### 2.4.1. sAA Level and Physiological Parameters

sAA was measured using a handheld biosensor (Salivary Amylase Monitor, Nipro Inc., Osaka, Japan). Briefly, the saliva collection strip was placed inside the mouth under the tongue for 30 s and then immediately inserted into the biosensor. After 30 s, the biosensor calculates the sAA level correctly in a linear range of 0–230 U/mL with a <9% coefficient of variation [19]. BP and HR were automatically displayed and recorded on the monitor (Dental Moneo BP-A308D; Omron Colin, Kyoto, Japan).

#### 2.4.2. Intensity of Anxiety and Pain

Anxiety and fear were assessed by the VAS-A, which was measured using a horizontal VAS line that ranged from 0 mm (“not at all anxious”) to 100 mm (“extreme anxiety”). The participants marked the point on the line that best represented their anxiety and fear during IV cannulation. The VAS score is determined by measuring (in mm) the distance of the mark from 0. The VAS-A has a significant relationship with other standard anxiety measures, including Corah’s Dental Anxiety Scale and Spielberger’s State Trait Anxiety Inventory. Therefore, it was considered to be a reliable indicator of anxiety in this study [20].

Pain intensity was assessed by the VAS-P, which was measured using a horizontal VAS line that ranged from 0 mm (no pain) to 100 mm (unbearable pain or worst pain imaginable), with higher scores reflecting greater pain intensity.

Patients were also monitored for adverse events, such as panic attack, nausea, or vasovagal reaction.

### 2.5. Statistical Analysis

The sample size was calculated using G*Power software 3.1 (Heinrich-Heine-University, Düsseldorf, Germany). Given that we intended to use repeated-measures analysis of variance (ANOVA) between factors with a power of 80% and an alpha of 5, and expected a high size effect, we calculated that a total sample size of 14 was needed.

The sAA, SBP, DBP, MBP, HR, VAS-A, and VAS-P data were tested for normal distribution and for homogeneity of the variances. Although normal distribution was verified, homogeneity of the variances was not. The data for sAA, SBP, DBP, MBP, and HR at baseline and at the time of intravenous cannulation according to whether clear-colored or green-colored glasses were worn are summarized as the median and interquartile range. The Wilcoxon signed-rank test was used to evaluate changes in those parameters between baseline and intravenous cannulation according to the color of the glasses worn. The Mann–Whitney *U*-test was used to compare the values of sAA, SBP, DBP, MBP, HR, VAS-A, and VAS-P between the two groups.

Median VAS-A and VAS-P scores (with the interquartile ranges) were calculated according to the color of the glasses worn, and the Mann–Whitney *U*-test was used to estimate differences in the VAS scores for anxiety and pain according to the color of the glasses. All statistical analyses were performed using IBM SPSS Statistics (version 24.0; IBM Co., Armonk, NY, USA). All *p*-values reported are two-tailed. A *p*-value < 0.05 was considered statistically significant.

## 3. Results

### 3.1. Patient Characteristics at Baseline

Twenty-four participants were analyzed in this study. Table 1 describes their demographic characteristics. The mean patient age (and standard deviation) was 40.9 ± 11.9 years.

### 3.2. sAA Level and Physiological Parameters

Table 2 shows the effects of green color exposure on the sAA level, BP, and HR at baseline and during catheter insertion. In the clear group, the sAA level increased significantly from 73.0 (54.3, 88.0) U/mL at baseline to 124.0 (84.8, 156.8) U/mL during PIC insertion (*p* < 0.001, Wilcoxon signed-rank test). In the green group, there was no significant change in the sAA level (79.5 (52.0, 127.3) U/mL at baseline and 92.5 (61.8, 141.5) U/mL during PIC insertion, *p* = 0.362). Moreover, the Mann–Whitney *U*-test showed a statistically significant between-group difference in the sAA level at PIC insertion (*p* = 0.025) but not at baseline (*p* = 0.143).

There was no significant change in SBP and MBP between at baseline and at the time of PIC in both groups. DBP in both groups significantly decreased from baseline to PIC. HR in both groups seemed to decrease from baseline to PIC but was not significant. The Mann–Whitney *U*-test showed no significant between-group difference in SBP, DBP, MBP, and HR.

### 3.3. Anxiety and Pain Intensity

**Figure 3:**
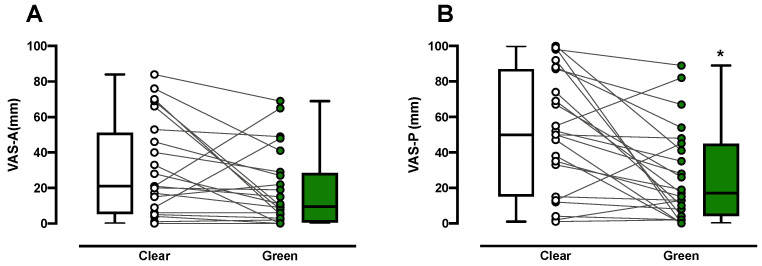
Self-evaluated anxiety and pain during peripheral intravenous cannulation using a visual analog scale for anxiety (VAS-A, 0, no anxiety; 10, worst imaginable anxiety) and for pain (VAS-P, 0, no pain; 10, worst imaginable pain) in the clear control group (*n* = 24) and green group (*n* = 23). Medians, quartiles, and ranges are represented by boxes with corresponding individual values on the VAS-A (**A**) and VAS-P (**B**). * *p* = 0.011.

The intensity of anxiety during PIC was less with green color exposure than with clear color exposure in 18/23 subjects, although it was not significant (median VAS-A, 9.5 versus 21.0; *p* = 0.109, Mann–Whitney *U*-test, Figure 3A). Similarly, the intensity of pain during PIC was significantly less in the green group than in the control group in 19/24 subjects (median VAS-P, 17.0 versus 50.0; *p* = 0.011, Mann–Whitney *U*-test, Figure 3B).

### 3.4. Adverse Events

No adverse events were observed.

## 4. Discussion

This study examined whether green color exposure could reduce stress, anxiety, and pain during catheterization in dental patients undergoing IV sedation. The main findings were as follows: the sAA level in the control group increased significantly from baseline during PIC but with no hemodynamic change, green color exposure suppressed the increase in sAA level during PIC, and green color exposure reduced VAS-P scores compared with no color exposure during PIC. These findings indicate that green color exposure attenuates stress and pain intensity in patients during PIC without any adverse effects. To our knowledge, this is the first study to examine the ability of green color exposure to reduce stress and pain during PIC in dental patients.

In this study, the sAA level in the control group increased significantly from baseline during PIC. sAA activity and physiological indicators, including SBP, DBP, MBP, and HR, were measured to assess the patient’s stress level. An increase in sAA reflects autonomic nervous system activity, including sympathetic nerve stimulation, which increases the total salivary protein concentration, and parasympathetic stimulation, which increases the salivary flow rate [21]. Therefore, sAA increases in a variety of situations, including painful conditions and dental stress [22,23,24,25,26,27]. Previous studies have found increases in sAA and perceived stress levels from baseline in patients subjected to the acute stress associated with venipuncture [21,28], which is in agreement with our results. Meanwhile, several other studies have also examined the association between hemodynamic changes and needle pain [29,30]. In those studies, there were no significant changes in hemodynamic variables in the control groups before and after PIC, which is consistent with our findings. Although only DBP in both groups decreased significantly from baseline during PIC in this study, the decrease might result from a vasovagal reflex-like reaction. Therefore, sAA activity might be a more sensitive marker of stress than BP or HR during PIC. Therefore, measurement of sAA could be useful for assessment of stress.

Green color exposure suppressed the increase in sAA during PIC. Recently, Ibrahim et al. reported that green light exposure (8 h/day for 5 days) induced not only antinociception and anti-allodynia but also an anxiolytic effect in rats via the visual system [15]. They also showed that green light improved pain and quality of life in patients with fibromyalgia and those with migraine [16,17]. The patients in those studies were exposed to green light for an average of 1.5 h/day for 10 weeks. Noseda et al. found that 3 min of green color exposure improved acute migraine attacks [31]. Furthermore, Hoggan et al. reported that blocking red or blue light by using coated glasses reduced pain intensity in patients with migraine [32]. Based on these studies, we evaluated the effects of short-term green color exposure on stress, anxiety, and pain levels during PIC by using green-colored glasses and obtained both objective and subjective measurements. Our findings suggest that green color exposure may suppress sympathetic activity due to anxiety and pain, resulting in inhibition of sAA.

Consistent with the sAA results, the VAS-A and VAS-P scores during PIC were lower in the green group than in the control group. In the green group, the VAS-A score decreased in 78.3% of patients and the VAS-P score in 82.6% (Figure 3). The mechanisms via which green color exposure reduces pain and anxiety are unknown. We used green color glasses in this study and found them to have antinociceptive effects similar to green light exposure. Therefore, the effects of green color may require the visual system rather than the integumentary system. An earlier animal study demonstrated that green light exposure increased enkephalins in the spinal cord and decreased entry of calcium through N-type voltage-gated calcium channels in sensory neurons, which have a strong relationship with antinociception [15]. These findings suggest that the antinociceptive effects of even short-term exposure to a green color are at least in part attributable to production of endogenous opioids. Taking our results together, the effects of green color exposure could be associated with the connection between the visual system and the pain modulation system [33].

It is also likely that wearing colored glasses is a “distraction technique” that attenuates anxiety and pain, similar to virtual reality methods or watching television [14,34]. In our preliminary study of 13 patients, we compared the effect of “wearing glasses” with that of wearing clear glasses and found no difference in all parameters among two groups (data not shown). A further study is needed to examine whether other colors can affect anxiety and pain during PIC.

The present study had several limitations. First, all study participants had a known history of fear or anxiety about dental treatment. Therefore, they might have been more sensitive and apprehensive about the prospect of a painful medical procedure, resulting in stronger responses. A further study might be needed to consider the patient characteristics. Second, we did not investigate the mechanism of the effects of green color exposure. More research may uncover an association between green color exposure and production of endogenous opioids or gamma-aminobutyric acid (GABA) [15,33]. Third, the investigators were not blinded to group allocation, which may have introduced some degree of bias leading to incorrect recording of results or an expectation about the treatment received. Finally, we did not examine the effects of other colors. We are presently embarking on a study of which color is best for reducing anxiety and pain during PIC.

## 5. Conclusions

In conclusion, wearing green-colored glasses is a safe way of reducing anxiety and pain during PIC. This simple, safe, and effective method may be useful when performing a painful dental procedure.

## Figures and Tables

**Table 1:** Patient demographic characteristics.

Patient Characteristics	Total (*n* = 24)	
Variables	Average	SD
Age (years)	40.9	11.9
Height (cm)	162.9	7.3
Body weight (kg)	63.0	13.6
Body mass index	23.8	4.8
Male/Female	10/14	
Dental phobia	18	
Gag reflex	6	

SD, standard deviation.

**Table 2:** Values for salivary alpha-amylase activity, blood pressure, and heart rate in patients in the clear group and patients in the green group.

	Clear Glasses				Green Glasses				*p*-Value ^b^
	Median	25th Percentile	75th Percentile	*p*-Value ^a^	Median	25th Percentile	75th Percentile	*p*-Value ^a^	
sAA (U/mL)									
Baseline	73.0	54.3	88.0	<0.001	79.5	52.0	127.3	0.362	0.143
PIC	124.0	84.8	156.8		92.5	61.8	141.5		0.025
SBP (mmHg)									
Baseline	128.5	118.3	139.8	0.213	135.5	125.8	148.3	0.772	0.288
PIC	129.5	120.3	155.0		133.5	117.0	154.0		0.951
DBP (mmHg)									
Baseline	86.0	81.3	97.3	0.005	88.5	81.0	95.0	0.010	0.788
PIC	77.5	72.0	93.3		82.5	74.3	91.3		0.703
MBP (mmHg)									
Baseline	99.0	93.1	111.0	0.248	106.3	94.5	112.8	0.095	0.477
PIC	98.5	82.5	110.8		95.8	89.3	113.8		0.710
HR (beats/minute)									
Baseline	81.0	76.3	91.8	0.072	80.0	69.5	91.5	0.174	0.672
PIC	74.0	69.3	88.8		77.5	69.3	89.0		0.516

sAA, salivary alpha-amylase activity; SBP, systolic blood pressure; DBP, diastolic blood pressure; MBP, mean blood pressure; HR, heart rate; PIC, peripheral intravenous cannulation; SD, standard deviation. ^a^ baseline vs. PIC, Wilcoxon signed-rank test. ^b^ Clear glasses group vs. green glasses group, Mann–Whitney *U*-test.

## Data Availability

The data presented in this study are available on request from the corresponding author. The data are not publicly available due to ethical restrictions.
